# Coronary Obstruction during Valve-in-Valve Transcatheter Aortic Valve Replacement: Pre-Procedural Risk Evaluation, Intra-Procedural Monitoring, and Follow-Up

**DOI:** 10.3390/jcdd10050187

**Published:** 2023-04-23

**Authors:** Francesca Romana Prandi, Yoav Niv Granot, Davide Margonato, Martina Belli, Federica Illuminato, Manish Vinayak, Francesco Barillà, Francesco Romeo, Gilbert H. L. Tang, Samin Sharma, Annapoorna Kini, Stamatios Lerakis

**Affiliations:** 1 Division of Cardiology, Mount Sinai Hospital, Icahn School of Medicine at Mount Sinai, New York, NY 10029, USA; francesca.prandi@mountsinai.org (F.R.P.); yoavgran@gmail.com (Y.N.G.); manish.vinayak@mountsinai.org (M.V.);; 2 Division of Cardiology, Department of Systems Medicine, Tor Vergata University, 00133 Rome, Italy; belli.martina@hsr.it (M.B.); fe.illuminato@gmail.com (F.I.); francesco.barilla@uniroma2.it (F.B.); 3 Cardiovascular Imaging Unit, San Raffaele Scientific Institute, 20132 Milan, Italy; margonato.davide@hsr.it; 4 Faculty of Medicine, Unicamillus-Saint Camillus International University of Health and Medical Sciences, 00131 Rome, Italy; romeocerabino@gmail.com; 5 Department of Cardiovascular Surgery, Mount Sinai Hospital, Icahn School of Medicine at Mount Sinai, New York, NY 10029, USA

**Keywords:** coronary flow, valve-in-valve TAVR, TAVR-in-TAVR, TAVR-in-SAVR, coronary obstruction, TEE, CT

## Abstract

Valve-in-valve (ViV) transcatheter aortic valve replacement (TAVR) is emerging as an effective treatment for patients with symptomatically failing bioprosthetic valves and a high prohibitive surgical risk; a longer life expectancy has led to a higher demand for these valve reinterventions due to the increased possibilities of outliving the bioprosthetic valve’s durability. Coronary obstruction is the most feared complication of valve-in-valve (ViV) TAVR; it is a rare but life-threatening complication and occurs most frequently at the left coronary artery ostium. Accurate pre-procedural planning, mainly based on cardiac computed tomography, is crucial to determining the feasibility of a ViV TAVR and to assessing the anticipated risk of a coronary obstruction and the eventual need for coronary protection measures. Intraprocedurally, the aortic root and a selective coronary angiography are useful for evaluating the anatomic relationship between the aortic valve and coronary ostia; transesophageal echocardiographic real-time monitoring of the coronary flow with a color Doppler and pulsed-wave Doppler is a valuable tool that allows for a determination of real-time coronary patency and the detection of asymptomatic coronary obstructions. Because of the risk of developing a delayed coronary obstruction, the close postprocedural monitoring of patients at a high risk of developing coronary obstructions is advisable. CT simulations of ViV TAVR, 3D printing models, and fusion imaging represent the future directions that may help provide a personalized lifetime strategy and tailored approach for each patient, potentially minimizing complications and improving outcomes.

## 1. Introduction

### 1.1. Transcatheter Aortic Valve Replacement

Aortic stenosis (AS) is the most frequent primary valve disease requiring surgery or a transcatheter intervention in Europe and North America [1], with an estimated prevalence of 3% in patients ≥75 years old [2,3] and an increasing incidence due to the aging of the world population [4]. Calcific degeneration represents the main etiopathogenesis in older patients. The natural history of AS is characterized by an initial latency period of a variable length, followed by the onset of symptoms that are mainly represented by the triad of angina, syncope, and dyspnea, which are associated with an unfavorable prognosis and a median survival of 1–3 years after their onset [5]. Both the American College of Cardiology/American Heart Association (ACC/AHA) 2020 guidelines [6] and the European Society of Cardiology (ESC) 2021 guidelines [1] recommend an intervention (class I indication) for symptomatic patients with severe high-gradient AS. 

The decision about the mode of this intervention (a transcatheter aortic valve replacement or TAVR versus a surgical aortic valve replacement or SAVR) follows the Heart Team’s careful evaluation. TAVR’s most common delivery approach is transfemoral. In randomized clinical trials (RCT), TAVR has been demonstrated to be superior to medical therapy in extreme-risk (inoperable) patients [7] and non-inferior to SAVR in high-risk [8,9] and intermediate-risk [10,11,12] patients at 5-year follow-ups. The Evolut Low Risk [13,14] and PARTNER 3 [15,16] trials have established TAVR, respectively, as non-inferior and superior to SAVR in low-risk patients at 2-year follow-ups. Recent data from the Evolut Low Risk trial confirmed the durable benefits of TAVR compared to surgery at 3-year follow-ups [17]. Numerous studies have shown that the transition from well-compensated hypertrophy to heart failure in patients with AS is led by myocardial fibrosis [18,19], which can be divided into diffuse fibrosis and replacement fibrosis; the latter occurs in a mid-wall distribution that can be detected by late gadolinium enhancement (LGE) on cardiac magnetic resonance imaging. Mid-wall replacement fibrosis is an independent predictor of mortality [20,21], and although this rapidly progressive scarring is arrested by an aortic valve replacement (AVR), it does not reverse, even two years after the AVR [22], representing an irreversible marker of LV decompensation in AS. This is the rationale of some ongoing randomized clinical trials, such as EVOLVED [23] and EARLY TAVR, that will provide further evidence for the potential long-term benefits of an early AVR in younger asymptomatic patients with severe AS.

In patients for whom a bioprosthetic aortic valve replacement is appropriate, a TAVR is currently recommended (class I indication), with a preference over SAVRs by the ACC/AHA 2020 guidelines, in patients who are >80 years old (or younger with a life expectancy of <10 years), with no anatomic contraindication to a transfemoral TAVR, with a high or prohibitive surgical risk with a life expectancy of >1 year, and with valve and vascular anatomy that is suitable for a transfemoral TAVR [6]. The ESC 2021 guidelines recommend a TAVR (class I indication) for patients that are ≥75 years old (this age cut-off is reduced compared to the ACC/AHA guidelines), or for those who are at a high risk (STS-PROM/EuroScore II > 8%) or unsuitable for surgery [1]. 

Key factors to consider during a Heart Team evaluation include the patient’s age, comorbidities, surgical risk scores, estimated life expectancy, and the prosthetic valve’s durability [1]. The potential need for a valve reintervention and the risks associated with this should be discussed with the patient [6]. Favorable TAVR anatomical characteristics represent an important feature to assess, including vascular anatomy (an accessible transfemoral delivery route and the absence of aortic root dilation) and valvular anatomy (the annulus size and shape, leaflet number and calcification, and coronary ostial height) [6].

A total of two valve types have been widely used for TAVRs, self-expanding valves (SEV) and balloon-expandable valves (BEV), based on the mechanism of the valve frame expansion. SEVs are generally supra-annular, resulting in a higher effective opening area (EOA), lower mean valve gradients, and lower rates of severe prosthesis–patient mismatch, so they are useful in patients with a small, calcific annulus and for TAVR-in-SAVR. A limitation of them is that, due to the higher frame height, access to the coronary ostia can be more challenging. BEVs are intra-annular, allowing for easier coronary access due to the shorter valve frame [24]. Still, few RCTs directly comparing SEVs and BEVs have been performed [25,26,27]. The CHOICE trial compared early-generation SEVs and BEVs and showed a higher device success with the BEVs at 30 days [25]; these results were not confirmed by a 5-year follow-up, and the BEVs were not associated with superior clinical outcomes, while the SEVs showed better forward-flow hemodynamics and lower rates of structural valve deterioration (SVD) [27]. The SOLVE-TAVI trial showed an equivalency between newer-generation SEVs and BEVs (including a coronary artery obstruction requiring an intervention) [26]. In addition to RCTs, large multicentric observational studies have been published that suggest a better performance of BEVs compared to SEVs [28,29,30]. Nevertheless, SEVs have been demonstrated to have better long-term outcomes in terms of moderate/severe SVD compared to a surgical bioprosthetic aortic prosthesis at a 6-year follow-up in low-surgical risk patients (NOTION RCT) [31], mainly due to the lower mean valve gradients present shortly after the procedure. Indeed, SEVs’ supra-annular leaflet position allows a larger EOA to be reached, so these results may not be generalizable to BEVs, which have an intra-annular leaflet position [31]. Ali et al. recently published data from the UK TAVI registry observing the stability of the hemodynamic function of TAVRs for up to more than 10 years of follow-up, with a low rate of severe SVD and valve-related death or reintervention, and a more frequent severe SVD with BEVs than SEVs [32]. Nevertheless, the possibility of reintervention on a valve bio-prosthesis must be considered, especially for younger candidates with a longer life expectancy. 

### 1.2. Valve-In-Valve (ViV) TAVR and Impact on Coronary Access and Coronary Obstruction Risk

The expansion of TAVR indication for younger, low-surgical-risk patients, along with the limited data on long-term (more than 10 years) bioprosthetic valve durability, will determine, in the next few years, the increase in patients that outlive their valve durability and need valve-in-valve (ViV) re-interventions for the management of a failed aortic bioprosthesis, mainly through TAVR-in-TAVR (also called Vi-TAVR or redo TAVR) and TAVR-in-SAVR (Vi-SAVR). TAVR-in-SAVR may be preferable to redo an SAVR in high-risk patients with a favorable previous prosthesis size, due to the lower permanent pacemaker implantation and dialysis rates that have been demonstrated [33]. In the current guidelines, ViV TAVR interventions have a class IIa recommendation for severely symptomatic patients with bioprosthetic valve stenosis/paravalvular regurgitation and a high or prohibitive surgical risk [6]. 

However, the TAVR procedure, both on native valves and even more on previous TAVRs or SAVRs, presents not-negligible risks of difficult coronary access and coronary obstruction. The need for a post-TAVR coronary angiography and revascularization for coronary artery disease (CAD) management is expected to increase with the aging of TAVR patients. Unfavorable coronary access after a TAVR occurs mostly with SEVs (supra-annular position) with taller valve frames, longer skirts, and smaller open cells: these characteristics reduce the opportunity to achieve commissure-to-commissure alignment with the native valve, which is essential to maintaining access to the coronary ostia [34,35]. Patients presenting with acute coronary syndromes following a TAVR have high in-hospital and late mortality rates, due to difficult coronary access and a lack of coronary revascularization [36]. The acute obstruction of a coronary ostium is a rare (an incidence of < 1%; it involves, in 90% of cases, the left coronary ostium) but devastating complication of TAVRs, with a 50% mortality rate at 30 days [37]. An acute coronary artery obstruction can be led by different mechanisms: the displacement of the leaflets of the native valve or previous transcatheter heart valve (THV) toward the coronary artery ostium or sino-tubular junction (STJ) during valve deployment, the positioning of the THV frame or commissural suture in front of the coronary ostium [38], a coronary embolic process from leaflet thrombosis [39], and flow variations in the Valsalva sinuses with a thrombus formation after a TAVR [40]. Female sex, a low coronary height (<10 mm), a shallow sinus of Valsalva (<30 mm), a virtual THV to coronary ostium distance (VTC) of <4 mm, and heavily calcified native leaflets are all features associated with an increased risk of coronary obstruction. Improper TAVR positioning (too high) is another risk factor for coronary obstruction. Vi-SAVRs present a four to six times higher risk of coronary obstruction (around 2.5–3.5% of cases) compared to TAVRs on native valves [37,41,42], since surgical valves are generally supra-annular, thus lowering the coronary height, and because valve suturing decreases the sinus width; the highest risk is in the case of TAVRs on stentless surgical bioprostheses or on stented bioprostheses with externally mounted leaflets [37,43]. TAVR-in-TAVR may cause a coronary obstruction due to sinus of Valsalva sequestration, if the degenerated THV leaflets are pushed against the STJ and maintained in the open position by the new THV; the Re-do TAVR registry showed a low risk (0.9%) of an acute coronary obstruction with a Vi-TAVR [44].

Considering the lifetime management of younger patients with a longer life expectancy, which may also require more than two aortic valve interventions, coronary flow obstruction must be considered in all three potential scenarios that may occur: SAVR-TAVR-TAVR, the most common scenario, which provides surgery at a young age and a later coronary access and coronary obstruction problem that only occurs when TAVR-in-SAVR or the subsequent TAVR-in-TAVR-in-SAVR are performed; TAVR-SAVR-TAVR, which has the benefit of a feasible fourth procedure (TAVR-in-TAVR-in-SAVR), but also the risks of TAVR explantation; and TAVR-TAVR-TAVR, which is completely minimally invasive but has an earlier risk of difficult access to the coronary arteries and coronary obstructions [38].

## 2. Effect of TAVR on Hemodynamics and Coronary Blood Flow

Contrary to the SAVR approach, in TAVRs, the native leaflets are preserved and expanded in the Valsava sinuses within the paravalvular space. TAVR implantation is therefore associated with major variations in the fluid dynamics inside the Valsalva sinus. Ducci et al. performed in vitro analyses of these fluid dynamics downstream from the valve, before and after a TAVR, documenting a reduction in the turbulence, velocity magnitude, and shear rate between the native valve leaflets and the aortic wall after the TAVR; the stagnation zone at the base of the sinuses may promote thrombus formation and contribute to thromboembolic events [40,45]. In vitro data with SEVs showed that a lower implant depth was associated with a better native sinus wash-out, but increased the neo-sinus stasis (due to a larger neo-sinus), which may prompt leaflet thrombosis; therefore, a higher implant position is preferable to reduce this neo-sinus flow stasis, although it is associated with an increased risk of coronary obstruction [46]. 

AS alters coronary physiology through changes in the blood flow input (due to reduced perfusion pressure through the narrowed valve, with an attenuated and delayed systolic forward compression wave) and output (the LV pressure overload leads to an increased myocardial mass that also contributes to an increased extravascular compression of the microcirculation and systolic coronary flow impedance, and consequent microcirculatory changes that are represented by the upregulation of the resting coronary blood flow, which prevents further upregulation and impairs the coronary flow reserve, CFR) [47]. In addition, the modulation of vasoactive factors contributes to the upregulation of the coronary blood flow at rest and endothelial dysfunction impairs hyperemic responses. AS causes a reduction in the coronary flow during systole, while the flow during the wave-free period of diastole is not affected, because during this time, the aortic valve leaflets are closed and do not contribute to the coronary flow, regardless of the severity of the AS [48]. A CFR impairment in AS may explain how AS can induce anginal symptoms despite unobstructed arteries: when the heart rate increases, the coronary diastolic suction wave decreases instead of increasing [49]. AVRs yield acute changes, represented by an increase in pulse pressure, mean arterial pressures, the magnitude of the systolic forward compression wave, and coronary perfusion pressure (due to the combined effect of increased forward-travelling pressure driving blood into the coronary and reduced backward-travelling pressure due to the compression of the microcirculation), and delayed changes, including CFR improvement, endothelial function restoration, and diastolic coronary perfusion wave recovery [47]. Improvements in the hyperemic myocardial blood flow and coronary vasodilator reserve after an SAVR were not directly related to left ventricular mass regression; they were attributed mainly to a reduced extravascular compression (with a consequently reduced systolic impedance to the coronary flow) and increased diastolic perfusion time [50]. CFR restoration after a TAVR was mainly driven by increased hyperemic blood flow rather than changes in the resting flow, and it was more significant in patients with a larger aortic valve area and greater LVEF increase after a TAVR [51]. The role of the valve orifice area in blood flow dynamics was also evaluated with four-dimensional (4D) flow cardiovascular magnetic resonance (CMR), an imaging technique that is capable of accurate flow visualization and quantification; an improvement in these blood flow dynamics after a TAVR procedure was especially seen when a larger effective orifice area index was obtained (EOAi) [52]. At this moment, there is scarce evidence on the effects of valve-in-valve TAVRs on the hemodynamic and coronary flows.

## 3. Coronary Hemodynamics in Patients with Severe Aortic Stenosis and Concomitant Coronary Artery Disease Undergoing TAVR

Coronary artery disease (CAD) is present in about half of TAVR candidates, with a decreasing trend because of the enrollment of younger and low-surgical risk patients, who have a much lower CAD prevalence [34]. Calcific aortic stenosis and CAD share predisposing factors such as older age, male sex, current smoking, a history of hypertension, and a high low-density lipoprotein (LDL) cholesterol. Moreover, early lesions of AS have several immunohistochemical features in common with coronary atherosclerosis [53,54].

Due to the impact of AS on the coronary blood flow, it may be challenging to accurately assess the severity of the coronary artery stenosis independently of the severity of the AS. A coronary angiography is the most reliable technique for ensuring CAD detection in TAVR candidates; other options include a computed coronary angiography (CTA) and pressure-derived indices of stenosis severity. CTA has an excellent negative predictive value and it may become an important tool for CAD screening in pre-TAVR work-ups, especially with the increasing number of low-risk patients [34]. During pre-procedural TAVR assessments, hyperemic indices of coronary artery stenosis that include systole (such as fractional flow reserve, FFR) are not reliable for assessing the severity of coronary artery stenosis, due to the lower microcirculation response to adenosine with a possible underestimation of the CAD extent, while indices restricted to the wave-free period of diastole (such as instantaneous wave-free ratio, iFR) are more accurate, as during this time, the flow is not influenced by the AS severity [48,55].

The indications for treatment, however, are still not entirely clear. In patients with severe CAD undergoing an SAVR, guidelines recommend performing concomitat coronary artery bypass grafting (CABG) [1], whereas there is no consensus on the management and timing of a percutaneous coronary intervention (PCI) for patients who are candidates for a TAVR. A meta-analysis comparing the outcomes of patients with severe AS and concomitant CAD, with and without a PCI prior/concomitant with a TAVR, found that pre-TAVR revascularization was not associated with increased 30-day or 1-year all-cause mortality rates [56]. The ACTIVATION (PercutAneous Coronary inTervention prIor to transcatheter aortic VALve implantaTION) trial demonstrated no difference in the primary composite end-points of all-cause mortality, rehospitalization at 1 year (but did not meet the noninferiority requirements,) and increased bleeding events in the PCI-prior to TAVR arm compared to the non-PCI arm [57]. In summary, no definite data exist on the optimal timing of a PCI for TAVR candidates with significant CAD, and a Heart Team evaluation is required to choose the correct timing of these PCIs on an individual basis. 

## 4. Pre-Operative Evaluation for Valve-In-Valve TAVR, How to Identify Patients at High Risk for Coronary Flow Obstruction and Preventive Strategies

Although the TAVR procedure has become an effective alternative to surgical replacement for patients with a failed surgical or transcatheter aortic prosthesis, patients undergoing ViV-TAVR treatment present more intraprocedural complications (including coronary obstructions) and a higher postintervention residual transaortic gradient compared to native valve transcatheter replacements [58]. Therefore, meticulous pre-procedural planning is crucial to minimizing the risks of intra- and post-procedural complications. 

**Figure 1:**
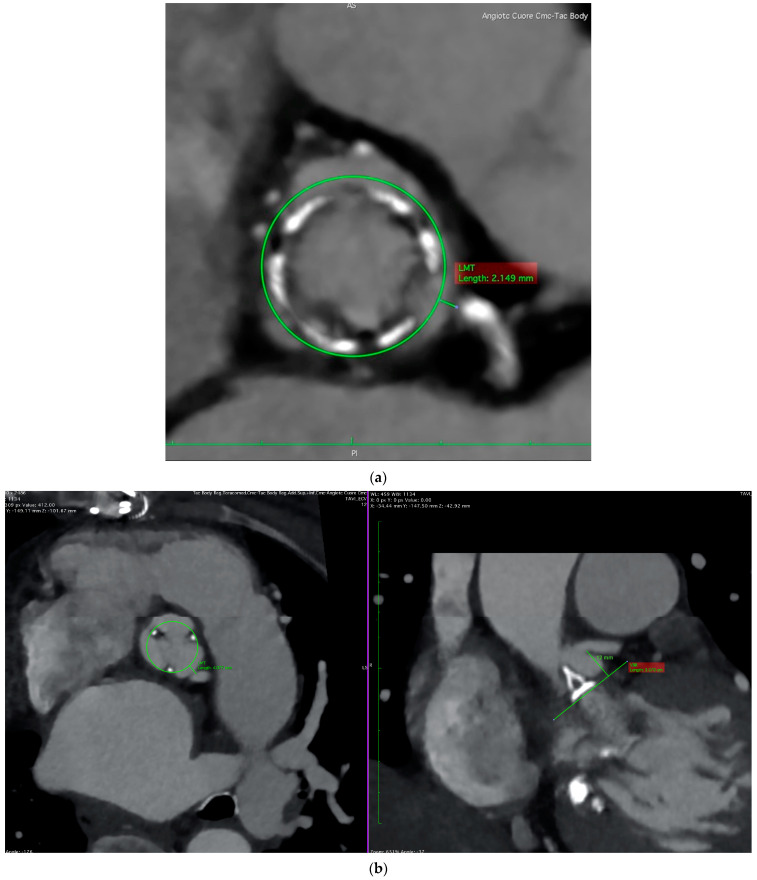
**Cardiac computed tomography.** Panel (**a**): valve to coronary (left main) distance < 4 mm, a typical case of a patient at high risk for coronary obstruction. Panel (**b**): although valve to coronary distance (4.08 mm) depicted this patient at intermediate risk for obstruction, the concomitant height of the coronary (12 mm) was adequate for safely performing ViV procedure.

The first step is to confirm the diagnosis of prosthesis dysfunction through transthoracic and transesophageal echocardiography (TTE and TOE, respectively). These examinations should also exclude other causes of increased transvalvular gradients that may represent contraindications to ViVs, such as leaflet thrombosis, significant paravalvular regurgitation, a patient–prosthesis mismatch, and active endocarditis. The second step is the sizing of the new implanted valve. As the new valve will be introduced inside the old one, the key measurement is the smallest internal dimension of the degenerated prosthesis [41]. Despite the helpful availability of valve-in-valve online applications [59], cardiac computed tomography (CCT) and three-dimensional echocardiography (for patients with a contraindication to CCT) are of the utmost importance, as the internal diameter of the degenerated prosthesis may differ from the pre-specified measurement of the manufacturer. The final and most important step during this pre-procedural planning is to identify the patients who are at a high risk of a coronary obstruction, as this is a potentially life-threatening complication that is far more common in patients treated with ViVs compared to those with a transcatheter replacement of their native aortic valve [42]. The main risk factors for obstruction are: low-lying coronary arteries (particularly the left main [58]), a shallow aortic root, supra-annular valves, large prosthetic leaflets, a stentless prosthesis (three-fold increased risk), and stented valves with externally mounted leaflets (six-fold increased risk) [37,42]. Taking all of these into account, it is self-explanatory why pre-procedural CCT is the gold-standard for assessing the risks of coronary obstruction sand planning intra-procedural strategies, due to its high spatial resolution. Indeed, the integration of CCT screening into ViV pre-procedural planning has already been shown to allow a reduction in coronary occlusion incidence during ViVs [60]. First, CCT can measure the coronary height, which is the distance from the coronary ostium to the aortic valve annulus: although there is no cut-off established for ViVs, there is general agreement to accept a cut-off of 12 mm distance, as is the case for transcatheter replacements of the native aortic valve [42,61]. The coronary height is less relevant for the coronary obstruction risks in ViVs than it is for native TAVRs; in ViVs, it is more important to evaluate the proximity of the coronary ostia to the anticipated final position of the displaced leaflets after a THV implant, and a low position of the coronary arteries will not cause a coronary obstruction unless the sinuses are shallow [61]. Patients with a narrow aortic root or low STJ have less space to contain the displaced prosthesis leaflets, whose dislodgment during valve expansion is the main mechanism involved in coronary obstruction, therefore inducing a higher risk of impaired blood flow toward the ostia. Post-TAVR CCT was shown to be useful in identifying the risks of coronary obstruction due to sinus sequestration in the case of a re-do TAVR; patients were considered at risk if the prior TAV commissure level was above the STJ and if the distance between the TAV and STJ was <2 mm for each coronary sinus [62]. Moreover, a risk of obstruction may be present in the case of a previously implanted bioprosthesis in a non-coaxial position, in relation to the long axis of the aortic root, despite its adequate dimension (typically >30 mm [58]). Another critical parameter to consider is the distance of the virtual valve to the coronary ostial, a measure obtained from an image in the transverse plane of the CCT re-elaborated in a specific image software, with knowledge of the size of the new prosthetic valve. A circular marker, created with this knowledge of the size of the new valve, is centered in the basal ring plane, and the distance from its edge to the coronary ostia is traced: a value < 4 mm is considered to be the prognostic cut-off for classifying a patient to be at a high risk for obstruction (see Figure 1) [37]. It is important to underline that these anatomic factors present a predictive role, even for the rare but possible complication of a delayed coronary obstruction [63]. Finally, stentless valves are at a higher risk for a coronary obstruction because their leaflets tend to expand outward after a new valve insertion without the three posts, which is typical of stented valves, limiting their movement [37]; however, in the case of previous aortic surgery, the operator may directly displace these valve posts toward the valve commissures, thus tarnishing their potential protective role in stented valves. 

Accordingly, appropriate pre-operative evaluations play a major role in the decision making of performing ViVs, along with an invasive strategy for protecting the coronary ostia with dedicated techniques. Preventive strategies include coronary protection with guidewires and an undeployed coronary balloon or stent positioned in the threatened coronary artery (Chimney stenting) [64,65,66]. The Bioprosthetic or Native Aortic Scallops Intentional Laceration to prevent Iatrogenic Coronary Artery Obstruction (BASILICA) technique has also been proposed as a safe and effective option for durably preventing a coronary obstruction in TAVRs and TAVR-in-SAVRs in patients at high risk [43,67], but it may not reliably prevent a coronary obstruction in TAVR-in-TAVR procedures, especially when Valsalva sinus effacement is the predicted mechanism of obstruction [68]. Indeed, in a TAVR-in-TAVR, the native leaflets remain in situ after the initial TAVR, acting as a barrier towards the left main orifice. The VIVID classification proposed a preprocedural CCT-based assessment of the BASILICA need for a ViV-TAVR for stented valves [69], but it is not applicable to stentless valves; a prospective study investigated the validity of the VIVID classification and found that coronary obstructions did not occur in any of the patients classified as high risk according to the VIVID classification [70]. The use of a transcatheter aortic valve that can be repositioned or retrieved in the case of a coronary obstruction following valve implantation is another option that can be considered for high-risk patients. ALIGN TAVR evaluated the impact of an initial transcatheter valve deployment orientation on the commissural alignment, demonstrating the importance of accurate pre-procedural planning in order to optimize the final valve alignment, avoid neo-a commissural overlap with the coronary arteries, and preserve the coronary access in a redo TAVR [71]. To achieve this goal, modified delivery systems for THVs have already been developed in order to obtain a better commissural alignment, but the results of this innovation in terms of a reduction in the difficulty of coronary access still need to be evaluated by larger studies. In addition, improvements in the hemodynamic outcomes from this approach have already been demonstrated [72].

## 5. Intra-Operative Monitoring of Coronary Flow during ViV-TAVR

An angiography of the aortic root can be very helpful in identifying the patients at risk of a coronary obstruction; the optimal projection should be perpendicular to both the surgical prosthesis and the coronary ostia, and since a left coronary obstruction is more common, a left anterior oblique (LAO) with cranial angulation is generally recommended. In the case of an inadequate aortic root angiogram (which is common in patients with a failed bioprosthesis, since aortic regurgitation causes quick contrast clearing from the aortic root), selective coronary angiography, especially of the left coronary, helps in assessing the coronary obstruction risk [61]. Pre-implant balloon valvuloplasty with a balloon size similar to that of the THV device can optimize the risk assessment for ostial coronary obstruction, since balloon inflation will determine the displacement of the bioprosthetic leaflets, similar to the one caused by a subsequent THV implant. Therefore, by injecting contrast above the inflated balloon, it will be possible to evaluate the flow into the coronary arteries, simulating the coronary flow scenario after a THV implantation [61]. During THV implantation, any movement of the coronary wire close to the coronary ostium (“wire sign”) may represent a warning sign for coronary occlusion [61]. Post-deployment aortography is generally useful for the diagnosis of coronary obstructions, but it may sometimes miss coronary obstruction detection; a TEE detection of new wall motion abnormalities may help as well.

**Figure 2:**
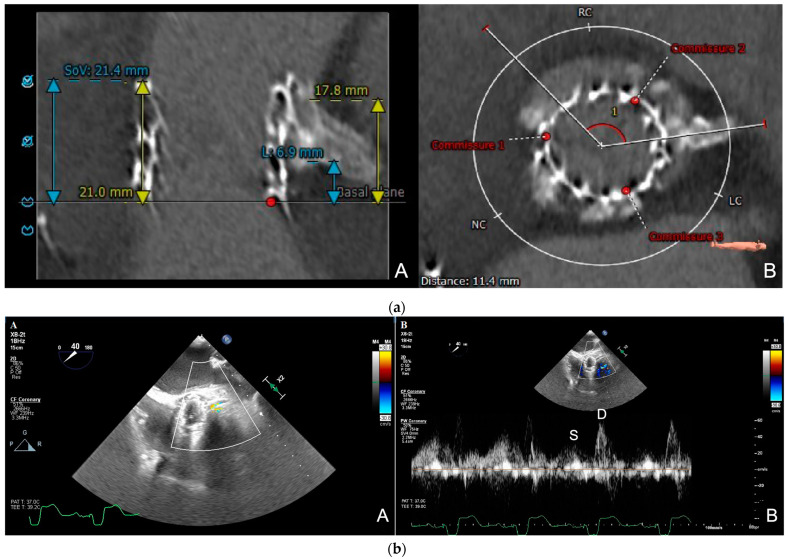
(**a**) **Cardiac computed tomography (CCT) scan.** Pre-procedural planning of TAVR-in-TAVR procedure in a symptomatic 85-year-old patient with 26 mm Sapien 3 (Edwards Lifesciences^TM^) valve structural degeneration and previous left main (LM) stent placement; CCT documented LM height of 6.9 mm (**A**) and Sapien 3 commissures in relation to both coronary arteries (**B**). (**b**) **Intraprocedural transesophageal echocardiography real-time monitoring of left main stent coronary flow during TAVR-in-TAVR procedure.** TAVR-in-TAVR procedure was performed under general anesthesia with intraprocedural TEE guidance; after pre-dilatation with a 24 mm True™ Dilatation Balloon (BD^TM^) to expand the valve frame, a 29 mm Evolut-FX valve (Medtronic^TM^) was deployed and finally balloon post-dilatation was performed to treat the presence of paravalvular leak, with excellent result. TEE after deployment documented diastolic flow through the LM stent with color Doppler (**A**) and normal systolic wave (“S”) and diastolic wave (“D”) velocities with pulsed-wave Doppler (**B**). There were no ischemic electrocardiographic changes during the procedure.

Generally, acute coronary obstruction during a TAVR presents with symptoms such as severe and persistent hypotension and electrocardiographic changes (mainly ST-T segment changes and ventricular arrhythmias), due to the fact that, in most cases, the left coronary artery is involved [37]. In these cases, a diagnosis can be confirmed through angiography, either by an aortogram or selective coronary catheterization [58]. However, sometimes these symptoms may be absent; silent coronary obstruction is probably an underdiagnosed complication of TAVRs and it may lead to a delayed coronary ischemia and sudden cardiac death after a TAVR [73]. In patients at a high risk for a coronary obstruction, transesophageal echocardiographic intra-operative monitoring of the coronary ostium flow represents a useful tool for detecting real-time coronary obstructions [73]. Several reports have shown that a TTE or TEE color-guided pulsed Doppler technique can measure the coronary flow velocity and that increased peak diastolic flow velocity can be used to detect significant coronary artery stenosis [74,75,76]. Intra-procedural TEE coronary flow monitoring was used during a TAVR procedure on a patient at risk for coronary obstructions, in terms of the coronary height and left coronary cusp calcification, and the detection of the coronary flow velocity acceleration at the left coronary ostium, from 66 cm/sec to 182 cm/s, a few minutes after the valve deployment, led to a silent left coronary ostium obstruction detection [73]. Although several reports have suggested that a local flow velocity of >2 m/s or a pre-stenotic to stenotic peak ratio of >2 have a good accuracy for detecting coronary artery stenosis [74,76,77], a cut-off value for the coronary flow velocity, as an indicator of the stenotic flow at the coronary ostium after a TAVR, has not been determined yet, as the coronary flow is affected by several hemodynamic factors, such as coronary perfusion pressure, which are modified after a TAVR, and technical issues, such as the scanning depth, angle, and quality of the pulse-wave Doppler. The post-to-pre TAVR peak ratio may be more reliable than the coronary flow velocity because it is less affected by anatomical and technical factors [77]. A single-center study observed that intraprocedural TEE monitoring of the left coronary artery (LCA) flow during a TAVR could be useful, especially for a silent, hemodynamically stable LCA obstruction detection, where the flow velocity was ≥0.9 m/s and the post-to-pre TAVR peak ratio was >2. Conversely, in patients with an unstable LCA obstruction, the flow velocity was <0.9 m/s and the post-to-pre peak ratio was <2, because hemodynamic collapse caused a reduced aortic and coronary perfusion pressure [77]. Therefore, intraprocedural TEE measurements of the left main coronary artery flow velocities, pre- and post valve deployment, are useful for detecting asymptomatic stable LCA obstructions, representing a promising tool for preventing delayed coronary obstruction. If a significant elevation of the velocity and/or velocity peak ratio is observed compared to the pre-procedural value, a selective coronary angiography is recommended. Intraprocedural TEE monitoring of the coronary flow may be particularly useful also for patients undergoing TAVRs or ViV TAVRs, which already have a stent in the left main (LM) coronary artery, to monitor, in real-time, the LM coronary patency (Figure 2, Videos S1–S3). 

The management of coronary obstructions during TAVRs or ViV TAVRs is generally achieved with PCIs, although hemodynamic support and conversion to open-heart surgery may be required in some cases [37]. Stent implantation may also be considered in cases of partial coronary obstructions, because of the risk of delayed adverse events; several factors should be considered, including the percentage of the diameter stenosis, the minimum lumen area, and the accelerated coronary flow [73].

## 6. ViV Post-Procedural Monitoring and Follow-Up of Patients at High-Risk for Coronary Obstruction

Although coronary obstruction as a TAVR complication generally occurs in the seconds or minutes after valve deployment [42], it may also develop as delayed coronary obstruction in the hours and days following the procedure (generally <24 h, but it is possible also in the months and years afterwards). It is associated with high in-hospital mortality rates [63]. Delayed coronary obstruction occurs more frequently with self-expanding valves than with balloon-expandable valves and is more common during ViV procedures; it can occur also in cases where an ostial coronary stent has been deployed during the procedure. Therefore, in patients at a high risk for coronary obstructions (a low coronary height, a narrow sinus of Valsalva, and ViVs, etc.), more intensive pre-discharge (such as longer monitoring in the intensive care unit [37] and a CCT before discharge) and post-discharge monitoring is advisable [63]. Indeed, a post-TAVR CCT is a useful tool for the early diagnosis of coronary obstructions in asymptomatic patients. 

## 7. Future Directions

For ViV TAVRs, coronary access is a key aspect, especially when considered from a lifetime perspective for young and low-risk patients [78]. CT simulations of TAVRs, followed by redo TAVRs, are able to predict whether a patient can undergo multiple TAVR procedures in their lifetime, estimating the risk of coronary obstructions related to sinus sequestration and the need for a leaflet modification technique; this tool may provide a personalized lifetime strategy for young patients with symptomatic severe AS [79]. 

3D printing and fusion imaging represent the future directions that may help in developing a tailored approach for each patient. Patient-specific 3D printed models have been used for TAVR and ViV TAVR pre-procedural planning to help with device sizing and the prediction of procedural complications, based on the specific patient’s aortic anatomy [80,81]. 3D printing has been used to assess the coronary artery obstruction risks during TAVRs and it has been found that 3D model simulations correlate well with clinical outcomes; therefore, 3D printing may represent a useful tool that may help in minimizing complications and leading to safer patient outcomes [82]. 

The fusion of 3D CCT-derived data with real-time procedural fluoroscopy during a TAVR procedure is feasible [83], provides an anatomic reference of the aortic root, including the aortic annulus, Valsalva sinuses, and coronary artery ostia [84], and allows for the display of prosthetic valve simulations at the most optimal implantation depths [85]. Therefore, CCT–fluoroscopy fusion imaging may help with optimal valve positioning and deployment [86,87] without the use of medium contrast (making it ideal for patients with renal function impairment) [85], and may potentially improve procedural outcomes [84], including the risk of coronary obstruction during TAVRs and ViV TAVRs. 

## Data Availability

No new data were created or analyzed in this study. Data sharing is not applicable to this article.

## Supplementary Materials

The following supporting information can be downloaded at: https://www.mdpi.com/article/10.3390/jcdd10050187/s1, Video S1—Initial aortogram documenting mildly under-expanded bioprosthetic valve and patent left main stent; Video S2—Transesophageal echocardiography (TEE) post TAVR-in-TAVR deployment showing stable position of the Evolut-FX valve (Medtronic^TM^) and left main stent; Video S3–Transesophageal echocardiography (TEE) post TAVR-in-TAVR deployment showing diastolic flow through the left main stent by color Doppler.
